# Autophagy-competent mitochondrial translation elongation factor TUFM inhibits caspase-8-mediated apoptosis

**DOI:** 10.1038/s41418-021-00868-y

**Published:** 2021-09-12

**Authors:** Chang-Yong Choi, Mai Tram Vo, John Nicholas, Young Bong Choi

**Affiliations:** Department of Oncology, Sidney Kimmel Comprehensive Cancer Center, Johns Hopkins University School of Medicine, Baltimore, MD 21287 USA

**Keywords:** Macroautophagy, Microbiology

## Abstract

Mitochondria support multiple cell functions, but an accumulation of dysfunctional or excessive mitochondria is detrimental to cells. We previously demonstrated that a defect in the autophagic removal of mitochondria, termed mitophagy, leads to the acceleration of apoptosis induced by herpesvirus productive infection. However, the exact molecular mechanisms underlying activation of mitophagy and regulation of apoptosis remain poorly understood despite the identification of various mitophagy-associated proteins. Here, we report that the mitochondrial translation elongation factor Tu, a mitophagy-associated protein encoded by the *TUFM* gene, locates in part on the outer membrane of mitochondria (OMM) where it acts as an inhibitor of altered mitochondria-induced apoptosis through its autophagic function. Inducible depletion of TUFM potentiated caspase-8-mediated apoptosis in virus-infected cells with accumulation of altered mitochondria. In addition, TUFM depletion promoted caspase-8 activation induced by treatment with TNF-related apoptosis-inducing ligand in cancer cells, potentially via dysregulation of mitochondrial dynamics and mitophagy. Importantly, we revealed the existence of and structural requirements for autophagy-competent TUFM on the OMM; the GxxxG motif within the N-terminal mitochondrial targeting sequences of TUFM was required for self-dimerization and mitophagy. Furthermore, we found that autophagy-competent TUFM was subject to ubiquitin-proteasome-mediated degradation but stabilized upon mitophagy or autophagy activation. Moreover, overexpression of autophagy-competent TUFM could inhibit caspase-8 activation. These studies extend our knowledge of mitophagy regulation of apoptosis and could provide a novel strategic basis for targeted therapy of cancer and viral diseases.

## Introduction

Mitochondria are required for a variety of cellular functions. However, an accumulation of dysfunctional mitochondria is harmful to cells, potentially via increased reactive oxygen species (ROS) and mis-localized mitochondrial DNAs that lead to oxidative stress, hyper inflammatory responses, or amplified apoptosis [1–4]. The quality control of mitochondria is achieved by balanced actions among mitochondrial biogenesis, dynamics, and mitophagy.

Mitophagy is a selective autophagy that removes dysfunctional or excessive mitochondria [5]. The mitochondria-localized kinase PINK1 and the ubiquitin (Ub) E3 ligase Parkin are the best known mitophagy-associated proteins. Upon the loss of mitochondrial membrane potential, the PINK1 and Parkin complex induces ubiquitination of mitochondria [6], which is recognized by Ub-binding autophagy receptors, such as p62/SQSTM1, and targeted to autophagosomes via interaction with autophagy-related protein 8 family proteins, such as LC3 and GABARAP. In addition, ubiquitination-independent mitophagy pathways via mitophagy receptors, such as NIX/BNIP3L, BNIP3, FUNDC1, and PHB2, have been identified under certain physiological conditions [7–10].

Mitophagy is also activated by virus infection, where it serves to counter host antiviral responses, such as type I interferon (IFN) induction and apoptosis, enabling successful virus infection [11–16]. We recently found that human herpesvirus 8 (HHV-8), also known as Kaposi’s sarcoma-associated herpesvirus, can activate NIX-mediated mitophagy via viral IFN regulatory factor 1 (vIRF-1) [17]. vIRF-1 appears to play important roles in blocking IFN and other stress responses, such as mitochondria-mediated apoptosis via BH3-only-proteins (BIM and BID) and MAVS (also known as IPS-1, VISA, and Cardif), to virus infection and replication through inhibitory interactions with cellular signaling proteins [18–21]. In studies to further understand the role of vIRF-1 in mitochondria, we identified the mitochondrial translation elongation factor Tu, encoded by the *TUFM* gene, as a vIRF-1 binding protein. TUFM is known to activate mitophagy upon virus infection [15, 22–24]. However, the exact localization of TUFM in mitochondria and role of TUFM in mitophagy have not been fully elucidated. Here, we characterize the role of TUFM in the inhibition of caspase-8-mediated apoptosis amplified by altered mitochondria and identify the existence of and structural requirement for autophagy-competent TUFM on the OMM.

## Results

### vIRF-1 binds directly to TUFM

**Fig. 1:**
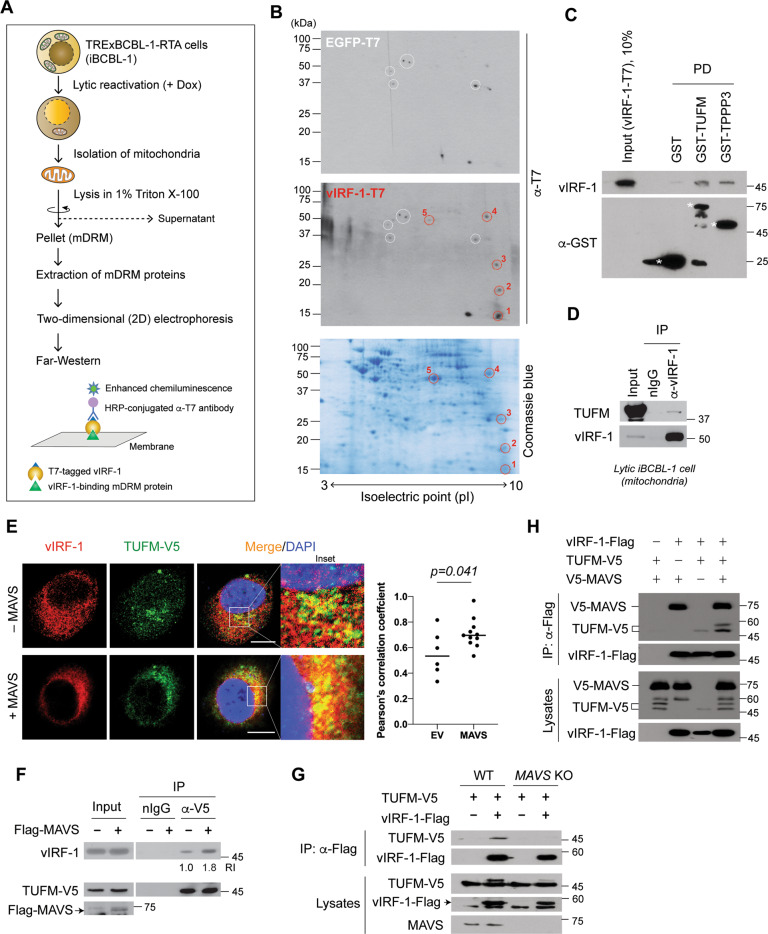
vIRF-1 binds directly to TUFM. **A** Workflow of Far-western blotting using the mitochondrial detergent-resistant microdomains (mDRM) isolated from iBCBL-1 cells treated with Dox for 2 days. **B** Two-dimensional (2D) separation of mDRM proteins and Far-western blotting using purified vIRF-1-T7 or EGFP-T7 protein. The third gel was stained with Coomassie blue. vIRF-1-reactive dots are marked by red circles on the Far-western blot and stained gel. As identified by mass spectrometry: dot 1, ATP synthase γ; dot 2, tubulin polymerization promoting protein 3 (TPPP3); dot 3, unidentified; dot 4, trifunctional enzyme subunit β, and dot 5, elongation factor Tu (TUFM). **C** Glutathione-S-transferase (GST) pull-down (PD) assay. Purified vIRF-1-T7 protein (250 ng) was incubated with GST, GST-TUFM, or GST-TPPP3, which were precipitated with glutathione beads. vIRF-1 and GST/GST-fusion proteins were detected by anti-vIRF-1 and -GST antibodies, respectively. Asterisks indicate GST and GST-fusion proteins of expected size. **D** Co-immunoprecipitation (Co-IP) of endogenous TUFM with vIRF-1. Mitochondrial extracts derived from lytic iBCBL-1 cells (Dox treatment for 2 days) were immunoprecipitated with pre-immune rabbit immunoglobulin (nIgG) or anti-vIRF-1 antibody, and then the precipitated complexes were immunoblotted with anti-TUFM or vIRF-1 antibody. **E** Indirect immunofluorescence assay (IFA) to assess co-localization of vIRF-1 and TUFM. HeLa.Kyoto cells were transiently co-transfected with vIRF-1 and TUFM-V5 in the presence or absence of Flag-MAVS for 24 h and immunostained with anti-vIRF-1 and -V5 antibodies. Note that the cells were permeabilized with 25 µg/ml of saponin for 5 min prior to fixation to diffuse out free vIRF-1 from the cytoplasm, thereby facilitating detection of mitochondria-bound vIRF-1 (see also Fig. S2). Intriguingly, the cell preparation enabled us to avoid detection of nuclear-localized vIRF-1, indicating that the mild detergent saponin could not permeabilize the nuclear envelope. The effect of MAVS on co-localization was analyzed using Coloc2 (ImageJ); the median Pearson’s correlation coefficients are 0.5339 and 0.6961 without and with MAVS, respectively. Scale bar, 10 µm. **F** Co-IP assay of vIRF-1-TUFM association. Total-cell extracts derived from 293T cultures co-transfected with vIRF-1 and TUFM-V5 in the presence or absence of Flag-MAVS for 24 h were immunoprecipitated with normal goat immunoglobulin (nIgG, negative control) or anti-V5 antibody, and then the precipitated complexes or lysates (input) were immunoblotted with anti-vIRF-1, V5, or Flag antibody. The relative intensities (RI) of coprecipitated vIRF-1 protein normalized to the immunoprecipitated TUFM-V5 are indicated. **G** Co-IP assay in wild-type (WT) and *MAVS* KO 293T cells transfected with the indicated plasmids. **H** Co-IP assay in 293T cells transfected with the indicated plasmids.

vIRF-1 localizes in part to mitochondria by targeting to the detergent-resistant microdomains (DRM) [21]. To examine the role of mitochondrial DRM (mDRM)-localized vIRF-1, we sought to identify vIRF-1-interacting mDRM proteins from the TRExBCBL-1-RTA (hereafter simply termed iBCBL-1) cell line, which is an HHV-8-infected primary effusion lymphoma (PEL) cell line with doxycycline (Dox)-inducible expression of RTA, a lytic switch protein [25]. Due to poor solubility of mDRM proteins in standard lysis buffer, we performed Far-western blotting instead of a co-precipitation method (Fig. 1A). Five distinct individual dots (Fig. 1B) highly reactive with vIRF-1-T7 compared to control EGFP-T7 were found, and the corresponding spots were excised from a replica gel for mass spectrometry analysis. One of the spots was identified as TUFM (Fig. S1).

An in vitro pull-down assay confirmed the direct interactions of vIRF-1 with TUFM and TPPP3, another hit protein (Fig. 1C). Furthermore, a co-immunoprecipitation (co-IP) assay showed that vIRF-1 interacted with endogenous TUFM on mitochondria isolated from lytic iBCBL-1 cells (Fig. 1D). In addition, an immunofluorescence assay (IFA) showed that vIRF-1 co-localized with TUFM in transfected HeLa.Kyoto cells, and their co-localization was promoted by overexpression of MAVS (Fig. 1E), which was reported previously to promote the mitochondrial targeting of vIRF-1 [21]. Consistent with this, co-IP assays showed that vIRF-1 interaction with TUFM was promoted by MAVS overexpression and greatly diminished by MAVS deficiency (Fig. 1F, G). However, vIRF-1 interaction with MAVS was not affected by TUFM overexpression (Fig. 1H), indicating that TUFM and MAVS interactions with vIRF-1 are not mutually exclusive.

### TUFM depletion potentiates apoptosis in lytic vIRF-1-deficient cells

**Fig. 2:**
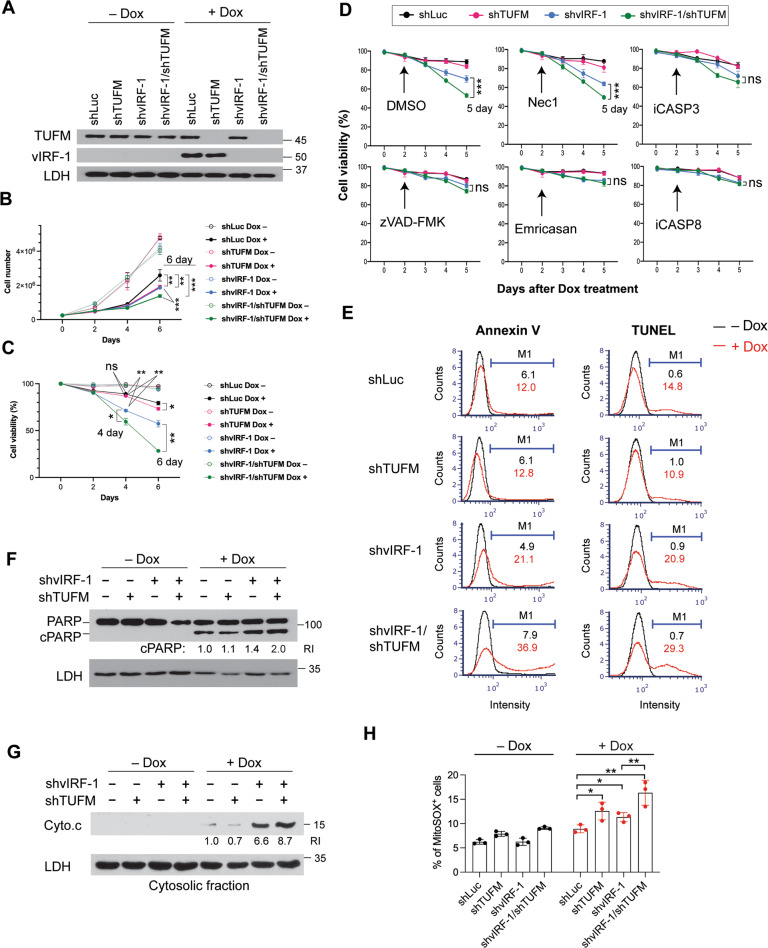
TUFM depletion potentiates apoptosis induced by vIRF-1 depletion. **A** Immunoblots of total-cell extracts derived from iBCBL-1 cells that were stably transduced with the indicated shRNAs and then left untreated or treated with 1 µg/ml Dox for 4 days to induce lytic replication. Lactate dehydrogenase (LDH) was used as a loading control. The iBCBL-1 cell lines were incubated with or without Dox for the indicated days, and cell proliferation and viability were measured using cell counting (**B**) and trypan blue exclusion (**C**, **D**) assays. Necrostatin-1 (Nec-1, 30 µM), the pan-caspase inhibitors zVAD-FMK (20 µM) and Emricasan (10 µM), z-DEVD-FMK (inhibitor of caspase-3, iCASP3, 2 µM), and z-IETD-FMK (inhibitor of caspase-8, iCASP8, 2 µM) were added to the cultures 2 days after Dox treatment (**D**). Particular datasets (bracketed) were analyzed by *t*-test for statistical significance. **E** The iBCBL-1 cell lines were left untreated or treated with Dox for 4 days and analyzed for apoptosis by annexin V staining and terminal deoxynucleotidyl transferase (TdT) dUTP nick end labeling (TUNEL). The proportion of apoptotic cells within each population was determined using an automated cell counter (Nexcelom), and the data were analyzed using FCS Express 6 software. M1 indicates the percentages of apoptotic cells. The total-cell extracts and cytosolic fractions derived from the iBCBL-1 cell lines left untreated or treated with Dox for 3 days were used for immunoblotting analyses of PARP cleavage (**F**) and cytochrome c release (**G**). The relative band intensities of cleaved PARP (cPARP) and cytochrome c normalized to LDH are noted under the corresponding bands. **H** Measurement of mitochondrial superoxide. The iBCBL-1 cell lines left untreated or treated with Dox for 3 days were incubated with 5 µM MitoSOX red reagent in Hank’s balanced salt solution. MitoSOX-positive cells were counted using the automated cell counter. The data present the mean ± SD of triplicate experiments. For **B**–**D**, **H**: *p < 0.05, **p < 0.01, ***p < 0.001, and ns not significant.

Mitochondria-localized vIRF-1 plays a crucial role for survival of lytically infected cells [17, 21]. So, we examined whether TUFM is involved in vIRF-1 regulation of cell survival using four different iBCBL-1 cell lines expressing Dox-inducible vIRF-1 and/or TUFM shRNAs (Fig. 2A). Luciferase shRNA (shLuc) was used as a control. Note that both lytic reactivation and shRNA expression are induced simultaneously by Dox treatment in the cell lines. Dox-inducible knockdown (KD) of TUFM and vIRF-1 proteins was verified using immunoblotting (Fig. 2A). As expected, vIRF-1-KD resulted in reduced numbers of iBCBL-1 cells after 4 days of Dox treatment (Fig. 2B). TUFM-KD also led to a significant decrease in numbers of shLuc and vIRF-1-KD lytic cells (Fig. 2B). To determine if the reduced numbers of TUFM-depleted lytic cells were associated with increased cell death, we counted the number of dead cells using a trypan blue exclusion assay. TUFM depletion indeed increased the proportion of dead cells within cultures of both cell lines, shLuc and shvIRF-1, during lytic replication; however, cell death was induced earlier in the vIRF-1-KD cells compared to in the shLuc cells (Fig. 2C). Overall, these results suggest that both vIRF-1 and TUFM are important for the survival of lytically infected cells.

We next determined the type of cell death promoted by TUFM depletion. Pan-caspase inhibitors zVAD-FMK and Emricasan, but neither DMSO nor the necroptosis inhibitor Necrostatin-1, inhibited cell death induced by TUFM-KD in lytic vIRF-1-KD cells (Fig. 2D). Specific inhibitors of caspase-8 (CASP8) and caspase-3 (CASP3) also inhibited the TUFM-KD-induced cell death (Fig. 2D), indicating that TUFM depletion is associated with caspase-dependent apoptosis. Indeed, TUFM-KD significantly promoted apoptosis in lytic vIRF-1-KD cells, as evidenced by Annexin V, TUNEL, and PARP cleavage assays (Fig. 2E, F). Moreover, TUFM-KD potentiated cytochrome c release to the cytosol in the vIRF-1-KD cells (Fig. 2G), suggesting that TUFM could inhibit mitochondria-mediated apoptosis. TUFM deficiency could lead to reduced mitochondrial respiratory activity and promote the production of ROS, which are probably involved in causing apoptosis. Indeed, mitochondrial superoxide was elevated significantly by TUFM-KD in not only lytic vIRF-1-KD, but also lytic control cells (Fig. 2H). However, TUFM depletion-induced oxidative stress is unlikely to be involved directly in apoptosis initiation because TUFM depletion alone was not sufficient to induce apoptosis (Fig. 2E–G). Together, these results suggest that TUFM may play a key and direct role in the inhibition of apoptosis in vIRF-1-deficient cells after lytic reactivation.

### TUFM inhibits CASP8 activation induced by dysregulated mitochondria

**Fig. 3:**
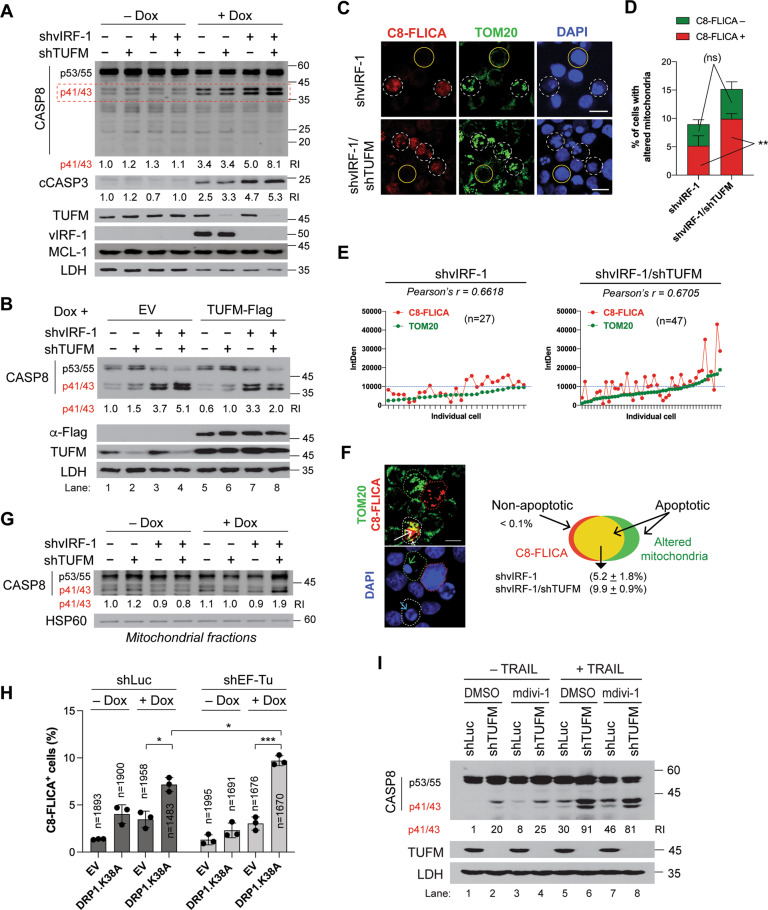
TUFM suppresses CASP8 activation induced by vIRF-1 depletion. **A** Immunoblots of total-cell extracts derived from the indicated iBCBL-1 cell lines that were left untreated or treated with 1 µg/ml Dox for 3 days. CASP8 cleavage products p41/43 (marked by the red dotted rectangle) in the different extracts were quantified relative to each other, after normalization to LDH; the values are provided below the CASP8 blot. In addition, the relative levels of the cleavage fragment (cCASP3) of CASP3, detected by antibody specific to cleaved CASP3, were determined as above and noted under the blot. **B** Immunoblots of total-cell extracts of the iBCBL-1 cell lines transduced with empty lentiviruses (EV) or lentivirus expressing TUFM-Flag and 1 day later treated with Dox for 3 days. The relative levels of the p41/43 fragments of CASP8 are noted under the blot. **C** Detection of CASP8 activity using SR-FLICA^®^ CASP8 (C8-FLICA) reagent in vIRF-1 KD or vIRF-1/TUFM dKD lytic iBCBL-1 cells treated with Dox for 3 days. After C8-FLICA staining, the cells were subjected to TOM20-IFA and DAPI nuclear staining. Scale bar, 15 µm. **D** Determination of the percentages of cells with altered mitochondria. Images were taken of four random fields for each culture: vIRF-1 KD (*n* = 570) and vIRF-1/TUFM dKD (*n* = 472). In addition, the percentage of C8-FLICA-negative or positive cells among cells showing altered mitochondria was determined. Statistical significance of vIRF-1 KD vs vIRF-1/TUFM dKD in C8 FLICA-negative or -positive cells was determined using student *t*-test. **p < 0.01; ns not significant. **E** The fluorescence intensities of TOM20 and C8-FLICA were measured in the cells with altered mitochondria above, vIRF-1 KD (*n* = 27) and vIRF-1/TUFM dKD (*n* = 47), using ImageJ software, and background-corrected integrated densities (IntDen) were plotted along with an arbitrary threshold (dotted blue line) of 10,000. **F** Functional relationship between altered mitochondria and CASP8 activation in the context of apoptosis. A representative image showing vIRF-1/TUFM dKD cells with either C8-FLICA (red dotted circle) or altered mitochondria (green dotted circle), or both (white dotted circle) after lytic reactivation is presented along with a DAPI image. White arrow indicates co-localization of C8-FLICA and mitochondria. Apoptosis was assessed by nuclear condensation (green arrow) or fragmentation (blue arrow). Scale bar, 10 µm. The chart shows the percentage of apoptotic vIRF-1 KD or vIRF-1/TUFM dKD cells with both C8-FLICA and altered mitochondria. It is noteworthy that the few cells (<0.1%) that are TOM20-negative and not nucleus-fragmented still showed CASP8 activation, supporting a requirement for mitochondria in CASP8-mediated apoptosis. **G** Immunoblots of mitochondrial extracts of the iBCBL-1 cell lines left untreated or treated with 1 µg/ml Dox for 3 days. HSP60 was used as a mitochondrial-protein loading control. The relative levels of the p41/43 fragments normalized to HSP60 are noted under the blot. **H** C8-FLICA assays in control (shLuc) and TUFM KD iBCBL-1 cell lines lentivirally transduced with EV or Flag-tagged DRP1^K38A^. The transduced cells were treated with or without Dox for 2 days, stained with C8-FLICA reagent, and subjected to Flag-IFA (Fig. S5). The percent of C8-FLICA-positive cells were determined and depicted in the graph. The data present the mean ± SD of three different images. *p < 0.05; ***p < 0.001. “*n*” indicates the total-cell numbers counted. **I** Immunoblots of cell extracts of the indicated shRNA-expressing HeLa.Kyoto cell lines left untreated or treated with 50 ng/ml of TRAIL and/or 20 µM mdivi-1 for 6 h. For induction of shRNA expression, the HeLa.Kyoto cell lines were pretreated with Dox for 3 days.

To define the mechanism of apoptosis promoted by TUFM depletion, we first examined caspase activation. Immunoblotting revealed that TUFM-KD promoted CASP8 activation, as evidenced by increased levels of the p41/43 fragments of CASP8, in lytic vIRF-1-KD cells, but not in lytic control cells (Fig. 3A). CASP3 was also activated but to a lesser extent (Fig. 3A). However, the levels of MCL-1, a key regulator of PEL cell survival [26], were not affected by depletion of either vIRF-1 or TUFM. Importantly, rescue with an shRNA-resistant version of TUFM substantially reduced CASP8 activation in lytic vIRF-1/TUFM-double-KD (dKD) cells (lane 8 in Fig. 3B). These results suggest that TUFM plays a key role in the inhibition of CASP8-mediated apoptosis induced by vIRF-1 deficiency.

As TUFM is implicated in mitophagy activation upon infection by certain RNA viruses [15, 27], we postulated that TUFM-KD leads to excessive accumulation of dysregulated mitochondria and thereby potentiates apoptosis in vIRF-1-deficient cells. To test this, we examined mitochondria using TOM20-IFA. The number of cells with both highly clustered mitochondria and apoptotic nuclei (fragmented or condensed chromatin) greatly increased in the vIRF-1/TUFM-dKD cells compared to vIRF-1-KD cells after lytic reactivation (Fig. S3). Emricasan inhibited nuclear fragmentation but not mitochondrial aggregation (Fig. S4), suggesting that mitochondrial aggregation precedes caspase-mediated apoptosis. Together, the results suggest that TUFM plays a key role in the inhibition of apoptosis via regulation of mitochondrial dynamics rather than direct inhibition of apoptosis-related caspases.

We examined the role of TUFM in the regulation of mitochondrial dynamics and apoptotic caspase activation using a combined approach of TOM20-IFA and CASP8 Fluorochrome Inhibitor of Caspases (C8-FLICA). C8-FLICA assay detects active CASP8 in cells. The number of vIRF-1/TUFM-dKD cells with both aggregated mitochondria and CASP8 activation was around 1.9-fold higher compared to vIRF-1-KD cells upon lytic reactivation (Fig. 3C, D). Intriguingly, the number of C8-FLICA-negative but still abnormal nuclear cells with aggregated mitochondria was equivalent between vIRF-1-KD and vIRF-1/TUFM-dKD cells (Fig. 3D), suggesting a selective role of TUFM in the inhibition of CASP8 induced by altered mitochondria. There was a modest but positive association between aggregated mitochondria and CASP8 activation in individual vIRF-1-KD and vIRF-1/TUFM-dKD cells (Fig. 3E). However, the dKD cells showed an increase in the number of cells with highly aggregated mitochondria together with intensive CASP8 activation compared to vIRF-1-KD cells (above the arbitrary threshold in Fig. 3E). Moreover, C8-FLICA co-localized in part with aggregated mitochondria in apoptotic cells (Fig. 3F), and the p41/43 fragments of CASP8 were detected at a higher level in the mitochondrial fraction of lytic vIRF-1/TUFM-dKD cells (Fig. 3G). Together, our results suggest that TUFM depletion-induced CASP8 activation is largely dependent on altered mitochondria.

To confirm that the proapoptotic effect of TUFM-KD is attributable to an accumulation of altered mitochondria, we transduced the control and TUFM-KD cells with DRP1^K38A^, a dominant negative form of DRP1 that inhibits mitochondrial fission [28]. Indeed, C8-FLICA assays showed that DRP1^K38A^ expression rendered the both cell lines highly sensitive to lytic reactivation-induced CASP8 activation but to a higher extent in TUFM-KD cells compared with control cells (Figs. 3H and S5). Similar results were obtained with mdivi-1 (Fig. S6), a mitochondrial division inhibitor [29]. Taken together, these results suggest that the proapoptotic effect of TUFM-KD in the context of vIRF-1 deficiency is associated with dysregulation of mitochondria quality control.

### TUFM can inhibit TRAIL-induced CASP8 activation in the OMM

Mdivi-1 is known to promote death receptor-mediated apoptosis through enhanced CASP8 activity in cancer cells [30, 31]. This synergistic effect appears to be associated with dysregulation of mitochondrial dynamics. Thus, we investigated whether TUFM depletion potentiates apoptosis induced by combined treatment with TNF-related apoptosis-inducing ligand (TRAIL) and mdivi-1. For this experiment, we generated HeLa.Kyoto cell lines expressing Dox-inducible shTUFM or shLuc. After Dox incubation for 3 days, the cells were treated with TRAIL and/or mdivi-1 for 6 h. Consistent with published findings, mdivi-1 promoted TRAIL-induced CASP8 activation in control cells (compare lane 5 with 7 in Fig. 3I). Interestingly, we found that mdivi-1 could not further enhance CASP8 activation in the TUFM-KD cells (compare lane 6 with 8 in Fig. 3I), while TRAIL greatly enhanced CASP8 activation in the TUFM-KD cells compared to the control cells treated with DMSO or mdivi-1 (compare lane 6 with lanes 5 and 7 in Fig. 3I). The negation of mdivi-1 activation of CASP8 by TUFM depletion and the efficacy of TUFM depletion alone on CASP8 activation in the context of these cervical cancer cells provides further evidence that TUFM functions to promote mitophagy and prevent the accumulation of damaged mitochondria.

### TUFM is localized on the OMM in modified and/or aggregate forms

**Fig. 4:**
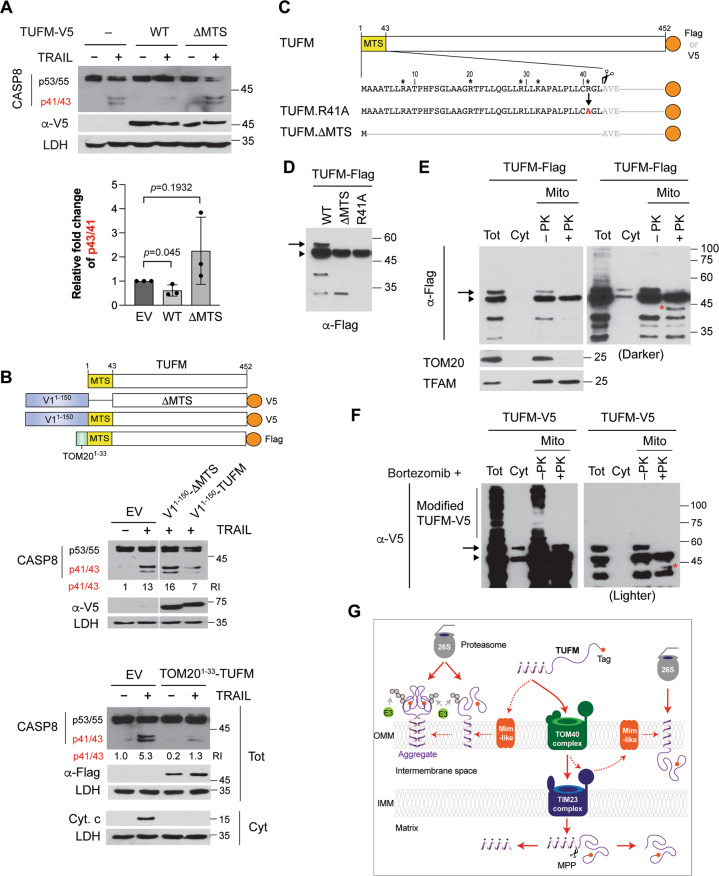
Structural determinants of OMM localization and associated anti-apoptotic function of TUFM. **A** Immunoblots of extracts of TUFM shRNA-expressing HeLa.Kyoto cells transfected with empty vector or wild-type (WT) TUFM-V5 or TUFM.ΔMTS-V5 expression plasmids and, 1 day later, treated with TRAIL, or mock treated, for 6 h. Transcripts encoding TUFM-V5 and TUFM.ΔMTS-V5 were resistant to TUFM shRNA. The graph presents the mean ± SD of three independent experiments. **B** Diagram of TUFM full-length and ΔMTS fused to the mDRM-targeting region (1–150 amino acids), V1^1–^^150^, of vIRF-1 or the N-terminal helix (1–33 amino acids), TOM20^1–^^33^, of TOM20. Immunoblots of extracts of HeLa.Kyoto cells transfected with the corresponding expression vectors and 1 day later treated with TRAIL, or mock treated, for 6 h. The relative levels of the p41/43 fragments of CASP8 normalized to LDH are noted under the blot. The cytosolic fraction of TOM20^1–^^33^-TUFM-transfected HeLa.Kyoto cells was used for Cyto. c and LDH immunoblotting. **C** TUFM preprotein and natural and mutated derivatives of TUFM involving the MTS sequence. Basic residues within the MTS region are indicated by asterisks. The TUFM.R41A variant was generated for experimental use (see below). The predicted cleavage site after the MTS is marked with a scissors image. Vectors were generated for expression of each of the indicated TUFM proteins, fused either to Flag or V5 epitope tag (orange circle). **D** Flag-immunoblot of extracts of transfected 293T cells expressing Flag-tagged TUFM WT, ΔMTS, or R41A. **E** Immunoblots of total-cell (Tot), cytosol (Cyt), and mitochondrial (Mito) extracts derived from TUFM-Flag-transfected 293T cells. Isolated mitochondria were treated with proteinase K (+PK) or left untreated (−PK). A darker blot is shown in the right panel. TOM20 and TFAM were used as markers of the mitochondrial outer membrane and matrix, respectively. **F** V5-immunoblots of total-cell extract and cytosolic and mitochondrial fractions derived from transfected HeLa.Kyoto cells expressing TUFM-V5 in the presence of 100 nM bortezomib. Mitochondrial fractions were treated or left untreated with PK. A lighter blot is shown in the right panel. The preprotein and cleaved (ΔMTS) forms of TUFM are indicated with an arrow and arrowhead, respectively. V5-reactive bands with molecular weights higher than the preprotein of TUFM are marked as “modified TUFM”. The red asterisk indicates a new band that appeared after PK treatment (**E**, **F**). **G** Predicted topological features of TUFM on the OMM. The presequence-carrying preprotein of TUFM is synthesized in the cytosol, imported by the translocase of the outer membrane (TOM40 complex) and the presequence translocase of the inner membrane (TIM23 complex), and cleaved by the mitochondria processing peptidase (MPP) in the matrix. In addition to translocation via the import pathway, the preprotein is localized to the OMM, facing outward towards the cytosol and presumably also internally (the band marked by red asterisks in **E**, **F**), potentially by a process involving an as-yet unidentified Mim-like protein. OMM-localized TUFM is subject to ubiquitination and proteasome-mediated degradation. Further details are mentioned in the Discussion section. E3 indicates a Ub E3 ligase.

We next examined the effect of TUFM overexpression on TRAIL-induced CASP8 activation. The TUFM variant (ΔMTS), which lacks the mitochondrial targeting sequences (MTS), was used as a control. Native TUFM, but not the ΔMTS form, inhibited CASP8 activation induced by TRAIL treatment (Fig. 4A). However, the inhibitory effect of TUFM was modest (~30–40% inhibition). As TUFM is mostly imported into the mitochondrial matrix, we hypothesized that a minority of TUFM protein on the OMM, particularly in mDRM, has an ability to inhibit CASP8. To test this, we enforced TUFM and TUFM.ΔMTS localization to mDRM by fusing them to the mDRM-targeting N-terminal sequence (V1^1–150^) of vIRF-1 (Fig. 4B). Proteinase K (PK) sensitivity and mitochondrial fractionation assays confirmed that the V1^1–150^ tag could drive TUFM to the cytosolic side and mDRM of the OMM (Fig. S7). Indeed, V1^1–^^150^-TUFM, but not V1^1–^^150^-TUFM.ΔMTS, could inhibit TRAIL-induced CASP8 activation by more than 50% compared to the empty vector control (Fig. 4B). Moreover, we found that the OMM-targeting sequence (amino acids 1–33) of TOM20 could drive TUFM to mDRM of the OMM (Fig. S7), and the fusion protein, TOM20^1–^^33^-TUFM, could significantly inhibit TRAIL-induced CASP8 activation (Fig. 4B). These results suggest that TUFM can inhibit CASP8 activation via the MTS and localization in mDRM of the OMM.

Since TUFM interacts with the cytosolic autophagy machinery, such as the ATG12-ATG5 conjugate, TUFM involved in autophagy activation and/or regulation of CASP8 is likely to locate on the cytosolic side of the OMM. As TUFM exists mostly in the cleaved (mature) form (ΔMTS), an OMM-localized TUFM is expected to comprise a preprotein that is larger than the cleaved form (Fig. 4C). Transfection experiments showed, as expected, that the major species produced from a wild-type TUFM vector was equivalent to the size of the ΔMTS protein (arrowhead in Fig. 4D), but additional bands, including a form migrating above the ΔMTS band (arrow in Fig. 4D), were detected upon prolonged exposure of the immunoblot. Mitochondrial processing peptidase (MPP) recognizes basic sequences of mitochondrial preproteins and cleaves a single site, often including arginine (R), at the −2 position[32]. Indeed, TUFM contains a putative MPP recognition arginine residue at position 41. To assess whether the slower-migrating band is the preprotein of TUFM, we generated the TUFM variant R41A by replacing the arginine with alanine (Fig. 4C). However, the size of TUFM.R41A was indistinguishable from that of TUFM.ΔMTS (Fig. 4D), indicating that R41 is not involved in MPP recognition and cleavage. It is unclear whether the upper band is the preprotein or a modified form of ΔMTS.

Protease sensitivity assays showed that the slow migrating TUFM band disappeared with PK treatment (Fig. 4E), indicating that it is located on the cytosolic side of the OMM. Intriguingly, a new TUFM fragment of about 40 kDa was detected after PK treatment (red asterisk in Fig. 4E), indicating that a Flag tag-containing C-terminal segment of the larger protein might be protected from PK digestion by the OMM with only a small region of the N-terminus exposed to the cytosol.

To investigate the possibility that there exists a form of TUFM orientated inversely with cytoplasmic exposure of containing the functional domains for regulation of autophagy or CASP8 activity, we used pharmacological inhibitors of protein degradation to potentially stabilize such a species. Amongst the tested inhibitors, only the proteasome inhibitors bortezomib and MG132 could stabilize the high-molecular-weight TUFM species observed previously (Fig. 4D, E) and other, slower-migrating forms (Fig. S8), which may represent aggregated and/or post-translationally modified forms of TUFM. Bortezomib-stabilized high-molecular-weight species present on isolated mitochondria were susceptible to PK digestion (Fig. 4F), providing evidence that they are located mainly on the cytosolic side of the OMM, but rapidly targeted for degradation mainly by the Ub-proteasome system. Taken together, we hypothesize that TUFM in part localizes to, and may aggregate within (Fig. S8), the OMM, with the C-terminus exposed to the cytoplasm, and thus is susceptible to cytosolic Ub modification and proteasomal degradation (Fig. 4G).

### TUFM is stabilized on mDRM upon mitophagy or autophagy activation

**Fig. 5:**
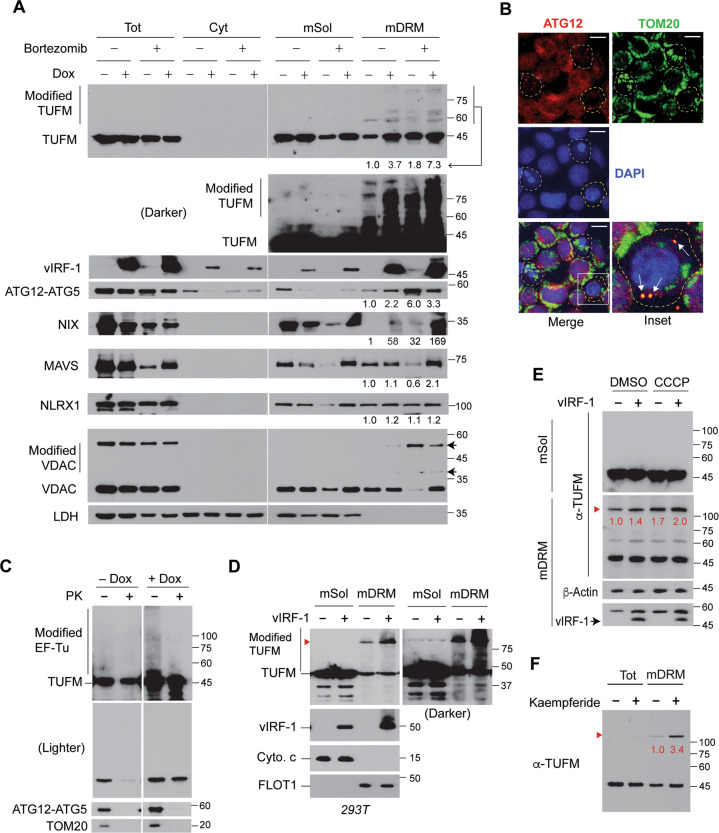
TUFM is stabilized on mDRM by vIRF-1, mitochondrial damage, and kaempferide. **A** Immunoblots of total-cell, cytosolic, and mitochondrial extracts of iBCBL-1 cells left untreated or treated with Dox for 3 days. Mitochondria were fractionated into detergent soluble (mSol) and insoluble (mDRM) fractions. To facilitate the detection of the modified TUFM proteins, the cultures were incubated with 100 nM bortezomib (proteasome inhibitor) for 1 day before cell collection. The ATG12-ATG5 conjugate was detected by anti-ATG12 antibody. Arrows indicate slow-migrating VDAC proteins, noted as “Modified VDAC”. A ratio was calculated for each blot by dividing the band intensity of mDRM-localized protein by the intensity of corresponding control band (no Dox and no bortezomib). VDAC and LDH were used as markers of mitochondria and cytosol, respectively. **B** IFA of ATG12 and TOM20 in lytic iBCBL-1 cells (Dox for 3 days). White dotted circles indicate apoptotic cells with low levels of ATG12 and altered mitochondria; the yellow dotted circle indicates a non-apoptotic cell, in which ATG12 co-localized with TOM20, indicated by arrows, and the level of mitochondria decreased. Scale bar, 10 µm. **C** PK sensitivity assays with mitochondria isolated from iBCBL-1 cells left untreated or treated with Dox for 3 days. The mitochondrial extracts were immunoblotted with anti-TUFM, ATG12, and TOM20 antibodies. Two exposures of the TUFM blots are shown. **D** Immunoblots of the mSol and mDRM fractions derived from 293T cells transfected with empty or vIRF-1 vector for 1 day. A darker TUFM blot is shown in the right panel. Cytochrome c (Cyto. c) and flotillin 1 (FLOT1) were used as makers of mSol and mDRM, respectively. Red arrowhead indicates TUFM bands of more than 100 kDa. **E** Immunoblots of extracts of 293T cells transfected with empty or vIRF-1 vector for 1 day and then treated with DMSO or CCCP (mitochondria protonophore) in the presence of 100 nM bortezomib for 6 h before cell collection. The relative levels of high-molecular-weight TUFM protein, normalized to β-Actin, are noted in red font below the bands. **F** Immunoblots of extracts of 293T cells treated with 20 µM kaempferide (TUFM-autophagy activator) for 1 day. The relative levels of the high-molecular-weight TUFM species normalized to the mature form are shown below the bands.

We examined whether endogenous TUFM can be detected in the modified/aggregated forms in virus-infected cells. Indeed, higher-molecular-weight TUFM proteins were readily detected in mDRM of lytic cells along with vIRF-1 and stabilized by bortezomib (Fig. 5A). Likewise, the ATG12-ATG5 conjugate and NIX, but not NLRX1, MAVS, and VDAC, also were more abundant on mDRM of lytic cells (Fig. 5A). Furthermore, ATG12-IFA showed that ATG12 could be localized to mitochondria (Fig. 5B). The modified TUFM and ATG12-ATG5 proteins on the mitochondria isolated from lytic iBCBL-1 cells were susceptible to PK digestion (Fig. 5C). Together, these results suggest that mDRM on the OMM may act as an assembly platform for TUFM-mediated autophagy.

We next examined the role of vIRF-1 in the modifications of TUFM using transfection. Indeed, vIRF-1 could promote TUFM modifications and/or aggregates in mDRM (Fig. 5D). Moreover, treatment with carbonyl cyanide *m*-chlorophenyl hydrazone (CCCP), a proton ionophore that triggers mitophagy [21], led to increased levels of the observed high-molecular-weight TUFM (>100-kDa) in both the absence and presence of vIRF-1, and to a more pronounced effect in the latter (Fig. 5E). Kaempferide, which is an inducer of TUFM-mediated autophagy [33], also enhanced the expression of mDRM-associated high-molecular-weight TUFM by 3.4-fold compared to the level in control cells (Fig. 5F). Together, our data correlate TUFM modification with autophagy activation and suggest that vIRF-1-effected TUFM modification may contribute to vIRF-1-promoted mitophagy in infected cells.

### TUFM activates autophagy via the canonical pathway

**Fig. 6:**
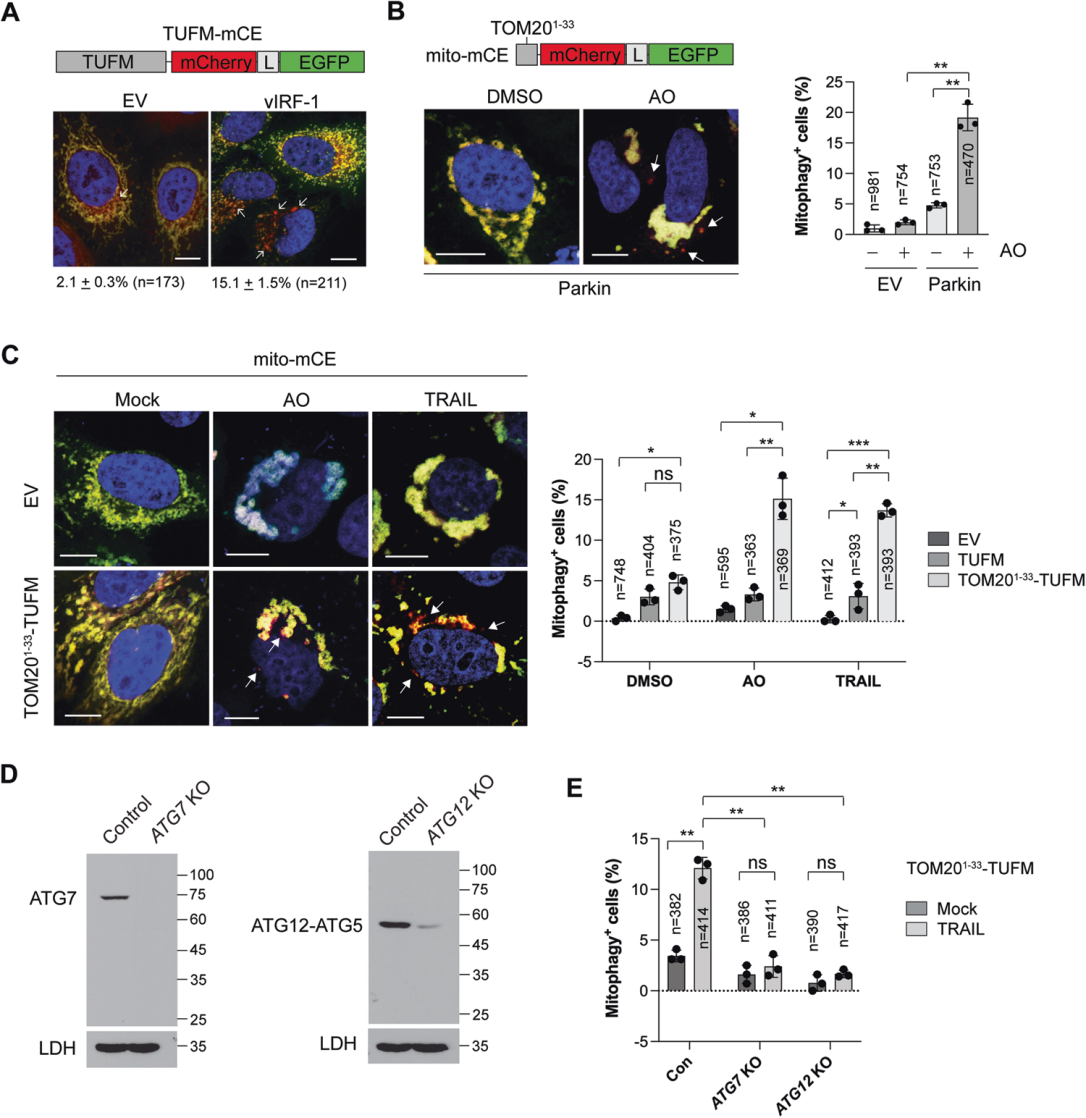
TUFM activates autophagy via the canonical pathway. **A** Confocal imaging of mitophagy with a tandem fluorescent-tagged TUFM (TUFM-mCherry-EGFP, termed TUFM-mCE). HeLa.Kyoto cells were transiently transfected with TUFM-mCE along with empty vector and vIRF-1. To facilitate the detection of mitochondria-containing autolysosomes, 10 µM leupeptin was added to the cultures. L indicates a glycine-serine linker. Arrows (red puncta) indicate the autolysosomes containing mitochondria. **B** Parkin-mediated mitophagy assay with a new mitophagy reporter, mito-mCE. The mCherry-EGFP tandem tag was fused to the MTS (amino acids 1–33) of TOM20. HeLa.Kyoto cells stably transduced with mito-mCE (HeLa.Kyoto^mito-mCE^) were transfected with empty or Parkin vector and 24 h later treated with DMSO or AO (2.5 µM antimycin A and 5 µM oligomycin) for 24 h in the presence of emricasan and leupeptin. **C** TUFM-mediated mitophagy assays. HeLa.Kyoto^mito-mCE^ cells were transfected with empty, TUFM, or TOM20^1–^^33^-TUFM vector and 24 later treated with DMSO, AO, or 50 ng/ml TRAIL for 6 h in the presence of emricasan and leupeptin. **D** Immunoblots of extracts of HeLa.Koyto cell lines stably expressing control, ATG7, or ATG12 gRNA along with Cas9. **E** TUFM-mediated mitophagy assays in control, *ATG7* KO and *ATG12* KO HeLa.Kyoto cell lines. The cell lines were co-transfected with TOM20^1–^^33^-TUFM and mito-mCE and 24 h later treated with 50 ng/ml TRAIL for 6 h. Representative cells (scale bar, 10 µM) are shown (**A**–**C**), and graphical data (**B**, **C**, **E**) present the mean ± SD of three different images; “*n*” indicates the number of cells counted. *p < 0.05; **p < 0.01, ***p < 0.001, and ns not significant.

To assess if vIRF-1 promotes TUFM-mediated autophagy flux, a tandem TUFM-mCherry-EGFP (TUFM-mCE) fusion protein was generated and transfected into HeLa.Kyoto cells with or without vIRF-1 (Fig. 6A). Once TUFM-mCE is delivered to lysosomes upon autophagy, the signal of EGFP, but not mCherry, is quenched. About 2% of TUFM-mCE-transfected cells showed punctate structures with only red fluoresce (basal autophagy), but vIRF-1 increased up to 15% the population of cells showing this pattern (Fig. 6A). To assess mitophagy flux, we generated a mitophagy reporter, mito-mCE, in which the tandem mCherry-EGFP tag was attached to the TOM20^1–^^33^ sequence for its mitochondrial targeting (Fig. 6B). The reporter worked for assessment of AO (antimycin A and oligomycin)-induced mitophagy in Parkin-transfected cells (Fig. 6B). The mitophagy assays using mito-mCE showed that TOM20^1–^^33^-TUFM, and to a lesser extent native TUFM, promoted mitophagy flux upon AO or TRAIL treatment (Fig. 6C). However, TOM20^1–^^33^-TUFM-medated mitophagy induced by TRAIL was abolished in *ATG7* or *ATG12* KO cells (Fig. 6D, E), indicating that TUFM-mediated mitophagy is activated via the canonical autophagy pathway.

### TUFM dimerization via the GxxxG motif within the MTS is required for autophagy

**Fig. 7:**
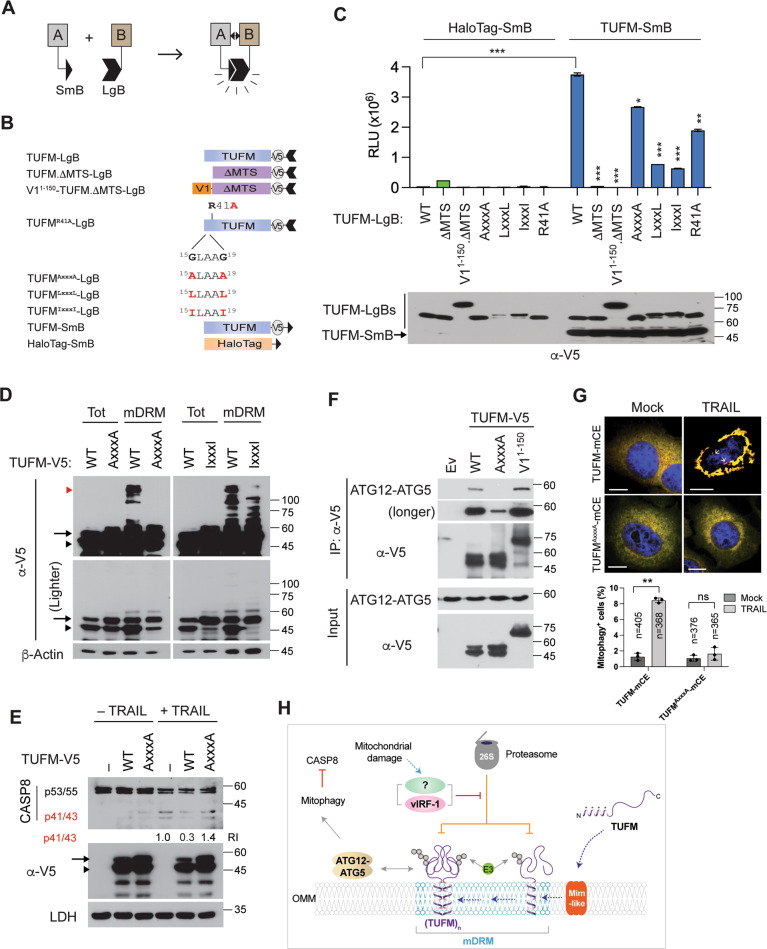
TUFM dimerization via the MTS is essential for autophagic inhibition of CASP8. **A** Principle of the NanoBiT-based protein fragment complementation assay. When a protein A interacts with its partner B, their fused Small BiT (SmB) and Large BiT (LgB) fragments are brought into proximity, which allows structural complementation thus yielding a functional enzyme acting on Nano luciferase substrate. **B** Schematic structure of the NanoBiT-fused proteins used in the following studies. **C** NanoBiT assays. 293T cells were transiently transfected with the indicated NanoBiT binary plasmids. Each value presents the mean ± standard deviation of triplicate samples from two independent experiments. The expression of the NanoBiT TUFM proteins was examined by V5-immunoblotting (lower panel). **D** Immunoblots of total-cell and mDRM extracts derived from 293T cells transfected with V5-tagged TUFM, TUFM^AxxxA^, and TUFM^IxxxI^. Arrow, arrowhead, and red arrowhead indicate the preprotein, cleaved form, and aggregated (or dimerized) form of TUFM, respectively. Lighter V5 blots are shown in the lower panels. **E** Immunoblot of extracts of HeLa.Kyoto cells transfected with empty vector (–) or TUFM-V5 or TUFM^AxxxA^-V5 expression plasmids for 24 h and treated with 20 ng/ml of TRAIL for an additional 6 h before cell collection. The relative intensities of the p41/43 fragments of CASP8 normalized to LDH are noted under the blot. **F** Co-IP assays. 293T cells were transfected with V5-tagged TUFM, TUFM^AxxxA^, or V1^1–^^150^-TUFM for 24 h in the presence of 10 µM kaempferide and 50 nM bortezomib, and the cell extracts were subject to V5-IP. Immunoprecipitated complexes and lysates (inputs) were immunoblotted with anti-ATG12 or V5 antibody. **G** TUFM-mediated mitophagy assays in HeLa.Kyoto cells transfected with TUFM-mCE and TUFM^AxxxA^-mCE and treated with or without 50 ng/ml TRAIL for 6 h in the presence of emricasan and leupeptin. The data present the mean ± SD of three different images; “*n*” indicates the number of cells counted. **H** A proposed model of autophagy-competent TUFM. The TUFM preprotein synthesized in the cytosol is inserted into the OMM by a putative Mim-like protein and rapidly ubiquitinated by a Ub E3 ligase for Ub-proteasome-mediated degradation. However, vIRF-1 or an unknown factor that is activated by mitochondrial damage can inhibit the degradation of TUFM by unidentified mechanisms, thereby stabilizing TUFM on mDRM and promoting interactions with the autophagy machinery, such as the ATG12-ATG5 conjugate. These molecular interactions in turn activate mitophagy to attenuate CASP8 activation. *p < 0.05, **p < 0.01, and ***p < 0.001 (**C**, **G**).

We examined whether TUFM homodimerizes, using NanoBiT protein fragment complementation assay[34] (Fig. 7A). The NanoBiT subunits, Large BiT (LgB) and Small BiT (SmB), were fused to the C-terminus of TUFM-V5 (Fig. 7B). To verify its true homodimeric interaction on mitochondria, TUFM.ΔMTS and the unrelated HaloTag protein were included as negative controls; V1^1–^^150^-TUFM.ΔMTS and N-terminal point variants (see below) of full-length TUFM fused to LgB were also included (Fig. 7B). Co-transfected with TUFM-V5-LgB, TUFM-V5-SmB exhibited stronger luminescence intensity compared to HaloTag-SmB (Fig. 7C). The luminescence intensity was also reduced to background levels by deletion of the MTS. V1^1–^^150^-TUFM.ΔMTS also could not bind to TUFM (Fig. 7C). These results suggest that the MTS is involved in TUFM dimerization.

The dimerization of membrane proteins is often mediated by their transmembrane helix and highly stabilized by GxxxG motif within the helix[35]. TUFM contains GxxxG motif, ^15^GLAAG^19^, within the MTS. To examine if the introduced GxxxG motif is involved in TUFM dimerization, we replaced the glycine residues with alanine (A), leucine (L), or isoleucine (I). The NanoBiT assays showed that the self-interaction of TUFM was diminished by the introduced mutations, and most substantially by LxxxL and IxxxI that present bulky hydrophobic side chains (Fig. 7C). It is noteworthy that the TUFM^LxxxL^ and TUFM^IxxxI^ variants showed reduced levels of the predominant ΔMTS species, probably due to an interference with import to the mitochondrial matrix. TUFM.R41A also showed reduced dimerization (Fig. 7C). These results suggest that the MTS contains primary-structure determinants of TUFM dimerization.

### TUFM dimerization is required for autophagy regulation of CASP8 activation

We examined the involvement of the GxxxG motif in TUFM aggregation. Immunoblotting revealed TUFM^AxxxA^, and to a lesser extent TUFM^IxxxI^, failed to form high-molecular-weight species (Fig. 7D). Moreover, TUFM^AxxxA^ failed to inhibit TRAIL-induced CASP8 activation (Fig. 7E), suggesting that TUFM dimerization may be required for the inhibition of CASP8 activation. Furthermore, co-IP assays showed that ATG12-ATG5 was co-immunoprecipitated with TUFM and V1^1–^^150^-TUFM, but to a lesser extent with TUFM^AxxxA^ (Fig. 7F). Importantly, TUFM^AxxxA^-mCE failed to mediate mitophagy upon TRAIL treatment (Fig. 7G). Taken together, we propose a model for autophagy-competent TUFM (Fig. 7H), in which newly synthesized TUFM preprotein is inserted into the OMM, through the action of a putative mammalian mitochondrial import (Mim) complex [36], where it is able to activate autophagy by interacting with the autophagy machinery. vIRF-1 or a cellular mitophagic factor could stabilize autophagy-competent TUFM by protecting it from Ub-proteasome-mediated degradation.

## Discussion

Our findings suggest that TUFM on the cytosolic side of the OMM may play a crucial role in the inhibition of CASP8 via autophagy activation. TUFM has been reported to interact with several mitophagy-related proteins. For example, TUFM is known to mediate autophagy by interacting with the mitochondrial protein NLRX1 upon virus infection [22]. However, NLRX1 is located mainly in the mitochondrial matrix [37], and its expression in mitochondria is unchanged during HHV-8 lytic replication (Fig. 5A). TUFM is also known to mediate mitophagy by interacting with PINK1 upon mitochondrial damage [38, 39]. Intriguingly, PINK1 phosphorylation of TUFM at serine 222 was proposed to restrict TUFM to the cytosol and inhibit mitophagy in a negative feedback manner [38]. However, it is unlikely that PINK1 regulation of TUFM-mediated mitophagy occurs because cytosolic TUFM was not detected in lytically infected cells even in the presence of proteasome inhibitor (Fig. 5A). Consistent with our previous finding [17], NIX was abundant in mDRM of lytic cells (Fig. 5A). It still remains unclear how vIRF-1 activates NIX-mediated mitophagy [17]. In this regard, it would be interesting to examine in future how vIRF-1 recruitment of the autophagy machinery via TUFM facilitates NIX-mediated mitophagy during lytic replication.

The mechanism by which TUFM is localized to the OMM remains elusive. In yeast, the precursors of proteins with an N-terminal signal anchor sequence are typically inserted into the OMM by the mitochondrial import (Mim) complex (Mim1/Mim2)[40]. However, the Mim complex has been found only in yeast so far; it has not been possible to identify a homolog in mammals or other species by simple sequence comparisons. Interestingly, recent functional studies with trypanosomes revealed that the OMM protein pATOM36 can replace the Mim complex in yeast, although it does not show any sequence similarity to the Mim proteins [41]. Thus, the functional analog may have arisen by convergent evolution. Identification of a mammalian functional counterpart of Mim involved in OMM targeting of TUFM would be of considerable significance.

TUFM is highly expressed in several cancers including glioblastoma, cholangiocarcinoma, and colorectal cancer (CRC) [42–44], and increased expression of TUFM is associated with shorter patient survival in CRC[45]. CRC is one of the most common cancers with TRAIL resistance. Thus, therapeutic targeting of TUFM-mediated autophagy might be clinically beneficial in CRC.

In summary, TUFM-mediated mitophagy appears to play an important role in host antiviral responses and carcinogenesis by downregulation of CASP8-mediated apoptosis. We found new, complexed and modified, forms of OMM-localized TUFM, which potentially are involved in promotion of mitophagy and consequent inhibition of CASP8 activation. In particular, TUFM dimerization seems to be required for CASP8 regulation. Thus, pharmacological or genetic targeting of TUFM dimerization, without disrupting its mitochondrial translation activity, would facilitate experimental dissection of the multiple functions of TUFM and could provide a basis for the development of selective antiviral and cancer therapies specifically targeting OMM-localized TUFM interactions and activity. Overall, our findings further understanding of the role of TUFM in connecting autophagy and apoptosis and have broader therapeutic implications with respect to virus infections and cancer.

## Materials and methods

### Cell culture

TRExBCBL-1-RTA (a gift from Dr. Jae U. Jung, herein termed iBCBL-1) and its derivative lines were cultured in RPMI 1640 medium (Quality Biological) supplemented with 15% heat-inactivated fetal bovine serum (FBS), stable L-alanyl-glutamine (Glutamine XL), and streptomycin and penicillin, at 37 °C and 5% CO_2_. 293T, HeLa.Kyoto (a gift from Dr. Ron R. Kopito), and their derivative cell lines were cultured in DMEM supplemented with 10% FBS and antibiotics. The cell lines were tested for mycoplasma contamination (R&D systems) and if necessary cultured in plasmocin™ treatment or prophylactic (InvivoGen). Transient transfection with plasmids was performed using GenJet version II (SignaGen Laboratories). For stable and doxycycline (Dox)-inducible expression of short hairpin RNAs (shRNAs), culture cells were lentivirally transduced with shRNA-specifying sequences in the presence of 10 µg/ml polybrene overnight, and stably transduced cells were selected by growing in the presence of 1 µg/ml puromycin or 400 µg/ml geneticin for more than 1 month, and pooled clones were collected. For detection of mitochondrial superoxide, cells were incubated with 5 µM MitoSOX red indicator (Invitrogen) in Hank’s buffered salt solution containing calcium and magnesium for 10 min at 37°C just before cell fixation.

### Lentivirus production

To produce infectious lentiviruses, 293T cells were co-transfected with the lentiviral vector together with the packaging plasmid psPAX2 and the vesicular stomatitis virus G protein expression plasmid pVSV-G at a ratio of 5:4:1. Two days later, virions were collected from the culture medium by ultracentrifugation in an SW28 rotor at 25,000 rpm for 2 h at 4 °C. The virion pellets were resuspended in an appropriate volume of phosphate-buffered saline (PBS) to achieve 100× concentration. Transduction titers of lentiviruses were determined in 293T cells in the presence of appropriate antibiotic to select transduced cells.

### Isolation of mitochondria

Pure mitochondria were isolated using Axis-Shield OptiPrep iodixanol (Sigma-Aldrich). In brief, latent and lytic iBCBL-1 cells were homogenized in buffer B (0.25 M sucrose, 1 mM EDTA, 20 mM HEPES-NaOH [pH 7.4]) with 50 strokes of a Dounce glass homogenizer and centrifuged at 1000 × *g* for 10 min. An aliquot of homogenate was used as total-cell extract. The supernatant was further centrifuged at 13,000 × *g* for 10 min. The pellet was collected as a crude mitochondrial fraction. For further enrichment, the pellet was resuspended in 36% iodixanol, bottom-loaded under 10% and 30% iodixanol gradients, and centrifuged at 50,000 × *g* for 4 h. The mitochondria were collected at the 10%/30% iodixanol interface. For isolation of mitochondrial detergent-resistant membrane microdomains (mDRM), enriched mitochondria were incubated in TNE buffer (50 mM Tris-HCl [pH 7.4], 150 mM NaCl, and 1 mM EDTA) containing 1% Triton X-100 on ice for 30 min, and centrifuged at 21,000 × *g* for 10 min. The supernatant was used as a soluble mitochondrial fraction, and the pellet was used as the mDRM fraction. The pellet was boiled in 1× sodium dodecyl sulfate (SDS) sample buffer for SDS-PAGE.

### DNA manipulation

All polymerase chain reaction amplification and site-directed mutagenesis including point and deletion mutations were performed using SuperFi™ DNA polymerase (Thermo Fisher Scientific). Subcloning of open reading frames and their derivatives into expression plasmids including pICE (a gift from Steve Jackson; Addgene plasmid #46960), pcDNA3.1(+) (Invitrogen), plenti.puro (a gift from Melina Fan; Addgene plasmid #74218), pGEX-4T-1 (GE Healthcare Life Sciences), and pTYB4 (New England Biolab). Plasmids were purified using ZymoPURE II Plasmid Midiprep kit for transfection (Zymo Research). The plasmids and oligonucleotides used in this study are listed in Tables S1 and S2, respectively.

### Immunological assays

Antibodies used in immunological assays including immunoblotting, immunoprecipitation, and immunostaining are listed in Table S3. For the preparation of total extracts, cells were resuspended in RIPA buffer (50 mM Tris [pH 7.4], 150 mM NaCl, 1% Igepal CA-630, 0.1% SDS, and 0.25% deoxycholate) containing a protease inhibitor cocktail and protein phosphatase inhibitors including 10 mM NaF and 5 mM Na_3_VO_4_ and sonicated using Bioruptor (Diagenode) for 5 min in ice water at a high-power setting (320 W). For immunoblotting, cell lysates were separated by SDS-PAGE, transferred to nitrocellulose or polyvinylidene difluoride (PVDF) membranes, and immunoblotted with appropriate primary antibodies diluted in SuperBlock™-PBS blocking buffer (Thermo Fisher Scientific). Following incubation with HRP-labeled Ig-specific secondary antibody, immunoreactive bands were visualized using enhanced chemiluminescent (ECL) substrates, such as Clarity (Bio-Rad) or SuperSignal™ West Femto (Thermo Fisher Scientific), on an ECL film. ImageJ software (NIH) was used to quantify the signal from immunoblots.

For immunoprecipitation (IP), total-cell or mitochondrial extracts were prepared in RIPA-B buffer lacking SDS and incubated with specific primary antibody at 4 °C overnight and incubated with protein G-agarose beads (Cell Signaling Technology) for an additional 3 h. For the IP of V5- or Flag-tagged proteins, anti-V5-agarose (Bethyl Laboratories) and anti-DYKDDDDK tag(L5) affinity gel (BioLegend) were used. Immunoprecipitated complexes were washed with RIPA-B buffer, followed by elution of bound proteins with 1× SDS sample buffer. If necessary, Clean-Blot™ IP Detection reagent (Thermo Fisher Scientific) was used to avoid detection of IgG used in IP assays. For detection of ubiquitinated proteins, an extra wash was performed using RIPA buffer supplemented with 1 M urea after IP, and nitrocellulose membrane was heat-activated by autoclaving at 121 °C for 30 min prior to blocking with 5% nonfat dry milk in PBS containing 0.25% Tween 20 (PBS-T).

For indirect immunofluorescent assay (IFA), cells grown on a coverslip (and transfected) were fixed in Image-iT™ fixative solution (Thermo Fisher Scientific) and permeabilized in 0.5% Triton X-100 in PBS. iBCBL-1 cells were attached on poly-L-lysine-coated coverslips. For certain experiments, cells grown and transfected on poly-L-lysine-coated coverslips were permeabilized with 25 µg/ml of saponin in PBS containing 100 mM potassium chloride for 5 min at room temperature before fixation. Following incubation with SuperBlock™-PBS blocking buffer for 1 h at room temperature, coverslips were incubated with primary antibodies, washed with PBS, and then incubated with appropriate fluorescent dye-conjugated secondary antibodies. Coverslips were mounted in ProLong™ Gold Antifade Mountant containing DAPI (Thermo Fisher Scientific) on glass slides, and cells were imaged by Zen software on a Zeiss confocal laser scanning microscope 700 with a ×20 or oil-immersion ×40 and ×63 objective. For co-localization analysis, images were randomly acquired under the microscope, and co-localization was scored using the ‘Coloc2’ tool of ImageJ. The integrated intensity (IntDen) of stained proteins were determined using ImageJ.

### Far-western blot and mass spectrometry

mDRM proteins were extracted in sample lysis buffer (9 M urea, 2 M thiourea, 100 mM DTT, 2% CHAPS (w/v), 60 mM n-octyl β-D-glucopyranoside, 0.5% Zoom carrier ampholyte (pH 3 to 10), and protease inhibitor cocktail), separated by isoelectric focusing over immobilized pH gradient strips (Invitrogen), and further separated by SDS-PAGE in the second dimension (2D). The 2D gel electrophoresis was conducted in triplicate: two gels were used for Far-western blotting, and the third for Coomassie blue staining. For Far-western blotting, mDRM proteins in the 2D gels were transferred to PVDF membranes, which were then blocked with 5% nonfat dry milk in Tris buffered saline containing 0.1% Tween-20 (TBS-T) and incubated with 1 µg/ml of purified recombinant protein vIRF-1-T7 or EGFP-T7 for 3 h at room temperature. After washes in TBS-T (3 × 10 min), the blots were incubated for 1 h with HRP-conjugated anti-T7 antibody. Reactive spots were visualized by ECL. The corresponding spots were excised from the Coomassie blue stained gel, digested by trypsin, and analyzed by matrix-assisted laser desorption/ionization-time-of-flight (MALDI-TOF) mass spectrometry (Voyager, Applied Biosystems). vIRF-1-T7 and EGFP-T7 proteins were expressed as chitin-binding domain-fusion proteins in BL21 derivative Rosetta cells (Novagen). The T7-tagged proteins were purified using chitin beads as previously performed [21].

### GST-precipitation assays

Recombinant glutathione-S-transferase (GST) and GST-fusion proteins were expressed in Rosetta cells and purified by standard methods. 1 µg GST or GST-fusion protein was incubated with 20 µl bed volume of washed glutathione sepharose-4B beads for 1 h at room temperature. After washing in binding buffer (PBS plus 1% Triton X-100), the protein-bead complexes were incubated with 1 µg recombinant vIRF-1 or 293T cell lysates containing vIRF-1-Flag proteins at 4 °C overnight, washed in binding buffer four times, separated on SDS-PAGE, and subjected to immunoblotting.

### Cell viability and apoptosis assays

Dead cells existing prior to the experiments were removed using Histopaque-1077 (Sigma) or Dead Cell Removal kit (Miltenyl Biotec). Freshly isolated intact iBCBL-1 cell lines were treated with 1 µg/ml Dox for the indicated times. Cell viability was then measured using trypan blue exclusion assays. For apoptosis assays, cells were analyzed by FITC-annexin V staining (BioLegend) or terminal deoxynucleotide transferase (TdT)-mediated dUTP nick labeling (TUNEL) assay kit (AAT Bioquest). All imaging and quantitation of apoptotic cells were performed using Cellometer Vision CBA Image Cytometry (Nexcelom), which employs four independent images for cell counting. FCS Express 6 Flow software (De Novo Software) was used for data analysis.

### Mitophagy flux assays

HeLa.Kyoto cells expressing mitophagy fluorescence reporters, TUFM-mCE and mito-mCE, were imaged using the ZOE fluorescent cell imager (Bio-Rad) for quantitative analysis. The number of total and mitophagy-positive cells from three randomly selected images were counted. Representative cell images were taken using Zeiss confocal laser scanning microscope 700 with oil-immersion ×63 objective.

### Reagents

Chemical reagents were purchased from the following companies: MG132 and bortezomib from Cell Signaling Technology; proteinase K, necrostatin-1, doxycycline, bafilomycin A1, leupeptin, antimycin A, oligomycin, and CCCP from Millipore Sigma; Liensinine from AK Scientific; mdivi-1 from Cayman Chemical; TRAIL from BioLegend; z-IETD-FMK (iCASP8) and zVAD-FMK from Enzo Life Sciences; AZ 10417808 (iCASP3) from ApexBio; emricasan from SelleckChem; furimazine from Promega.

### Quantification and statistical analysis

Statistical analyses were performed using Prism 8 software (Graphpad Software, Inc.). Statistical significance is stated in the Figure legends and Supplemental Figure legends. Differences between controls and samples were determined by paired *t*-tests (two-sided) and were considered significant when the *p* value was less than 0.05 (*p* < 0.05).

## Supplementary information

Supplemental Materials

## Data Availability

All relevant data are available from the authors upon reasonable request.
