# Supplementary material for: Autophagy-competent mitochondrial translation elongation factor TUFM inhibits caspase-8-mediated apoptosis
Source: Cell Death Differ. 2021 Sep 12;29(2):451–64. doi: 10.1038/s41418-021-00868-y (PMC8817016; doi:10.1038/s41418-021-00868-y)
Supplement: Supplementary file 1 — Supplemental Materials [file 41418_2021_868_MOESM1_ESM.docx]

Supplementary Materials

**Fig. S1**

**
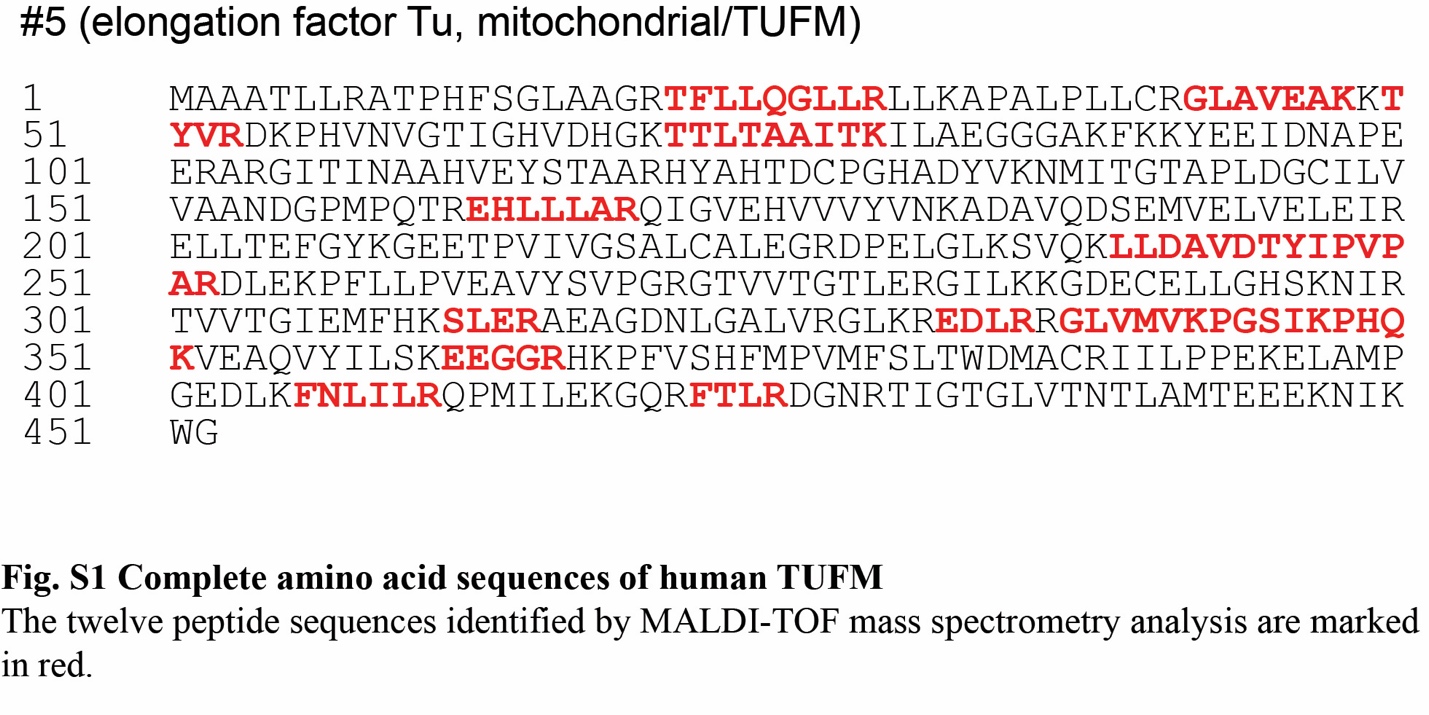
**

**Fig. S2**

**
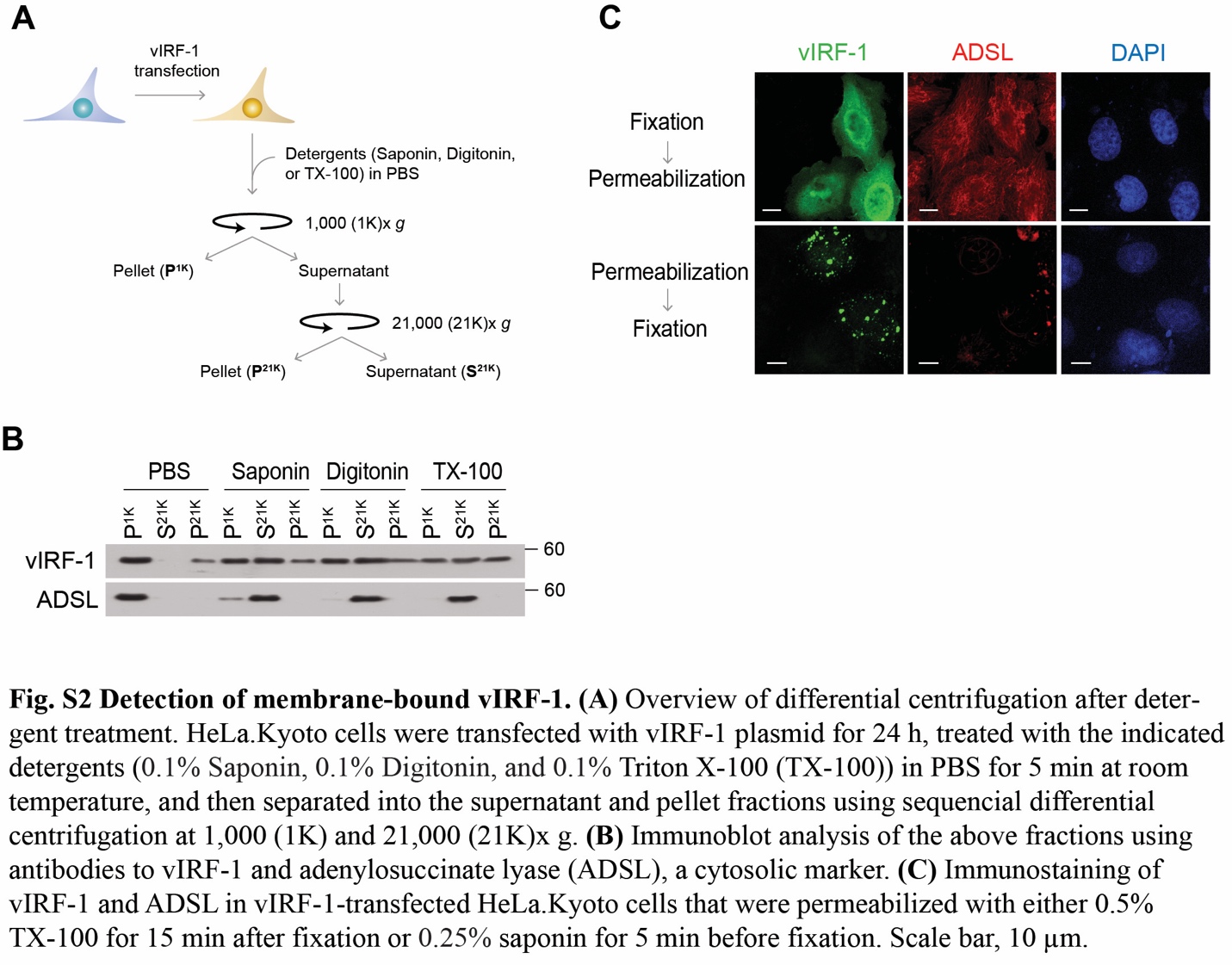
**

**Fig. S3**

**Fig. S4**

**
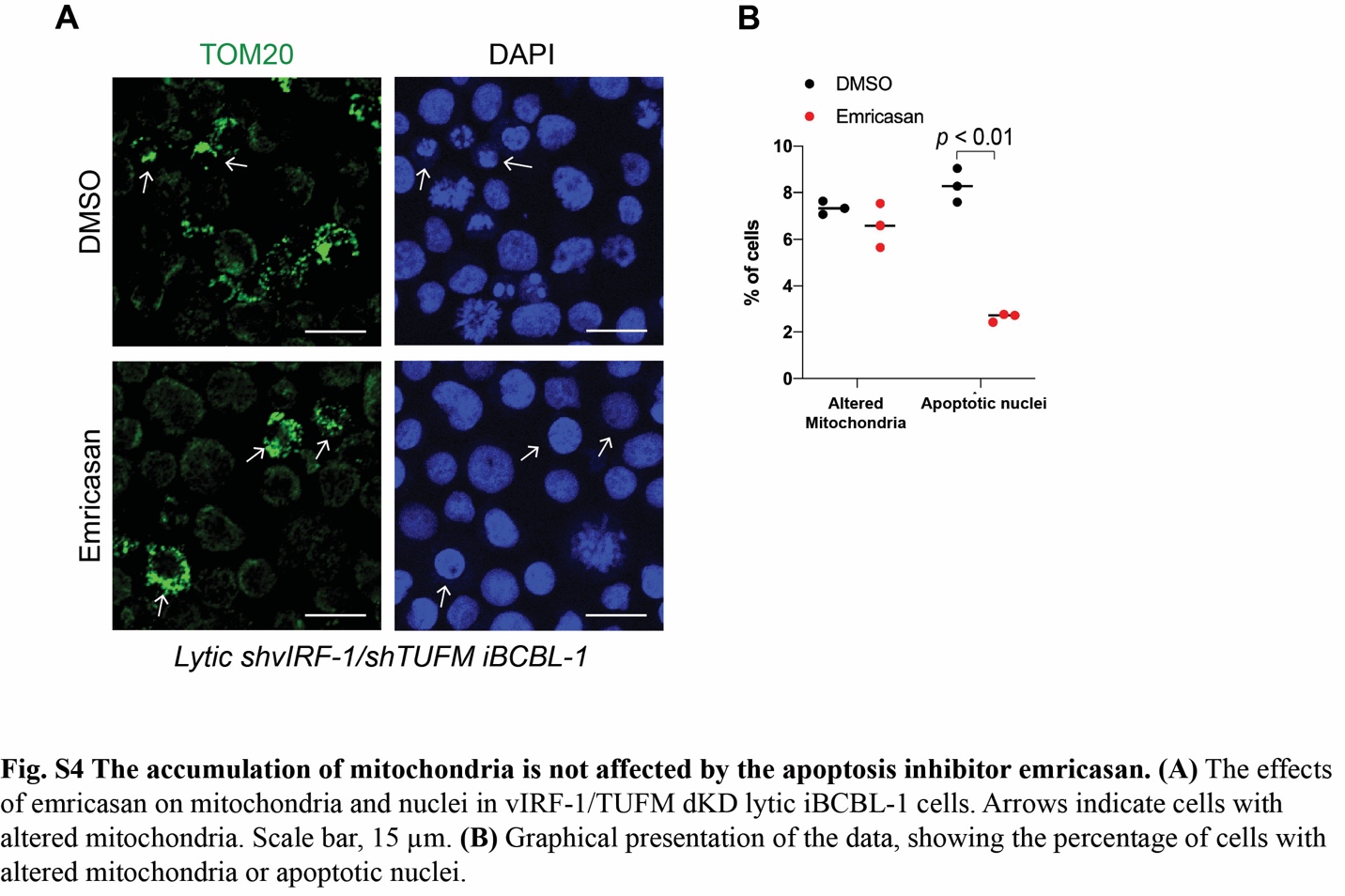
**

**Fig. S5**

**Fig. S6**

**
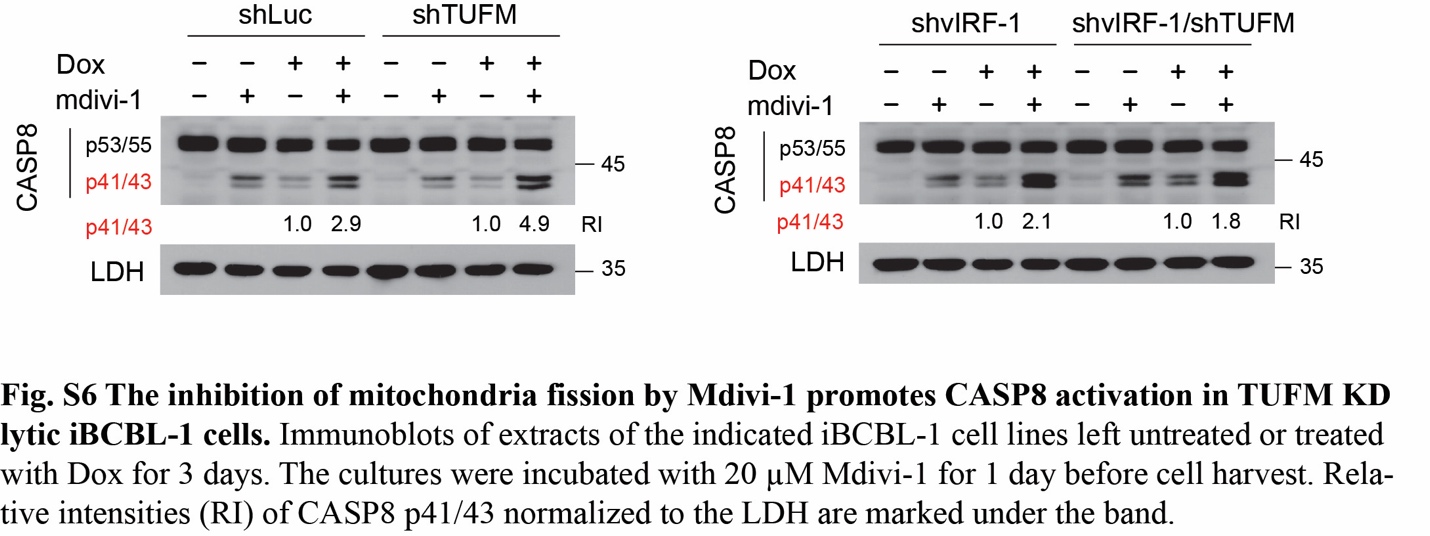
**

**Fig. S7**

**Fig. S8**

**Table S1 Plasmids used in the study**

| **Name** | **Source/Cloning or Mutagenesis** | **Identifier** |
| --- | --- | --- |
| pTYB4 | New England Biolab | E6901 |
| pTYB4_T7 | Lab stored (cloning T7 using EcoRI and SmaI) | N/A |
| pTYB4_EGFP-T7 | Cloning EGFP using NheI and EcoRI | N/A |
| pTYB4_vIRF-1-T7 | Cloning vIRF-1 using NheI and EcoRI | N/A |
| pGEX 4T-1 | Amersham | 27458001 |
| pGEX 4T-1_TUFM | Cloning TUFM using BamHI and XhoI | N/A |
| pGEX 4T-1_TPPP3 | Cloning TUFM using BamHI and XhoI | N/A |
| pICE | A gift from Steve Jackson | Addgene 46960 |
| pICE_V5 | Lab stored (Vo et al., 2019) | N/A |
| pICE-V5-MAVS | Cloning TUFM using BamHI and XhoI | N/A |
| pFLAG-MAVS | A gift from Edward Harhaj | N/A |
| Myc-vIRF1 | A gift from Jae Jung | N/A |
| Tet-pLKO-Neo | A gift from Dmitri Wiederschain | Addgene 21916 |
| Tet-pLKO-Neo_shTUFM | Cloning TUFM shRNA2 using AgeI and EcoRI | N/A |
| Tet-pLKO-Neo_shLuc | Cloning shLuc using AgeI and EcoRI | N/A |
| pcDNA3.1(+) | Invitrogen/ Thermo Fisher Scientific | V79020 |
| pcDNA3.1_Flag | Lab stored | N/A |
| pcDNA3.1_vIRF-1-Flag | Lab stored | N/A |
| pcDNA3.1_TUFM-Flag | Cloning TUFM using BamHI and EcoRI | N/A |
| pcDNA3.1_TOM20^1-33^- TUFM-Flag | Insertion of TOM20^1-33^ into pcDNA3.1_TUFM-Flag using HindIII and BamHI | N/A |
| pcDNA3.1_TUFM.ΔMTS-Flag | Cloning TUFM.ΔMTS using BamHI and EcoRI | N/A |
| pcDNA3.1_TUFM.R41A-Flag | Site directed mutagenesis | N/A |
| pcDNA3.1_TUFM-V5 | Replacing Flag with V5 tag using EcoRI/XhoI | N/A |
| pcDNA3.1_V1^1-150^-TUFM-V5 | Insertion of V1^1-150^ using BglII/BamHI in the BamHI site of pcDNA3.1_TUFM-V5 | N/A |
| pcDNA3.1_V1^1-150^-TUFM.ΔMTS-V5 | Insertion of V1^1-150^ using BglII/BamHI in the BamHI site of pcDNA3.1_TUFM-V5 | N/A |
| pcDNA3.1_TUFM^AxxxA^-V5 | Site directed mutagenesis | N/A |
| pcDNA3.1_TUFM^LxxxL^-V5 | Site directed mutagenesis | N/A |
| pcDNA3.1_TUFM^IxxxI^-V5 | Site directed mutagenesis | N/A |
| pICE_Flag | Lab stored (Vo et al., 2019) | N/A |
| pICE_V1^1-150^-TUFM.ΔMTS-Flag | Cloning V1^1-150^-TUFM.ΔMTS using HindIII and MluI | N/A |
| plenti.puro_(HA-Ub) | A gift from Melina Fan | Addgene 74218 |
| plenti.puro II | A derivative of plenti.puro (lab stored) | N/A |
| plenti.puro II_Flag | Cloning Flag tag using SalI and XbaI | N/A |
| plenti.puro II_TUFM-Flag (resist to sh2) | Cloning using EcoRV and SalI | N/A |
| plenti.puro II_DRP1^K38A^-Flag | Cloning using AgeI and SalI  (DRP1.K38A was amplified from pcDNA3-Drp1K38A) | N/A |
| pcDNA3-Drp1^K38A^ | A gift from Richard Youle | Addgene 45161 |
| pICE_TOM20^1-33^-mCherry-EGFP (mito-mCE) | HindIII-TOM20^1-33^-BamHI-mCherry-EcoRI-GS linker-MluI-EGFP-XbaI | N/A |
| plenti.puro II_TOM20^1-33^-mCherry-EGFP | Transferring mito-mCE in pICE using AgeI and XbaI | N/A |
| pICE_TUFM- mCherry-EGFP | Replacing TOM20^1-33^ in pICE_mito-mCE with TUFM (WT or AxxxA) using HindIII and BamHI |  |
| pBiT 1.1C_TUFM-V5 WT and variants | Cloning using BglII and XhoI | N/A |
| pBiT 2.1C_TUFM-V5 | Cloning using BglII and XhoI | N/A |
| HaloTag-SmB (negative control) | Promega | #2014 |
| psPAX2 | A gift from Didier Trono | Addgene 12260 |
| VSV-G | Lab stored | N/A |
| pRK5-HA-Parkin | A gift from Ted Dawson | Addgene 17613 |

**Table S2 Oligonucleotides used in the study**

| **Name** | **Forward or**  **Reverse** | **Sequences (5’ to 3’)** |
| --- | --- | --- |
| TUFM.R41 | F | GGCATTGCCTCTCTTGTGCGCCGGCCTGGCCGTGGAGGCC |
|  | R | GGCCTCCACGGCCAGGCCGGCGCACAAGAGAGGCAATGCC |
| TUFM res to shRNA2 | F | CATGCCCGGGGAAGATCTAAAATTTAATCTGATCTTGCGGCAGCCAATG |
|  | R | GCCGCAAGATCAGATTAAATTTTAGATCTTCCCCGGGCATGGCAAGCTC |
| TUFM^AxxxA^ | F | CCACTTCAGCGCACTCGCCGCCGCACGGACCTTCCTGCTGCAG |
|  | R | GGAAGGTCCGTGCGGCGGCGAGTGCGCTGAAGTGGGGCGTCGC |
| TUFM^LxxxL^ | F | CCACTTCAGCATTCTCGCCGCCATCCGGACCTTCCTGCTGCAGGGTC |
|  | R | GGAAGGTCCGGATGGCGGCGAGAATGCTGAAGTGGGGCGTCGCGCGC |
| TUFM^IxxxI^ | F | CCACTTCAGCCTTCTCGCCGCCCTCCGGACCTTCCTGCTGCAGGGTC |
|  | R | GGAAGGTCCGGAGGGCGGCGAGAAGGCTGAAGTGGGGCGTCGCGCGC |
| shLuc | F | CCGGTCCTAAGGTTAAGTCGCCCTCGCTCGAGCGAGGGCGACTTAACCTTAGGTTTTTG |
|  | R | AATTCAAAAACCTAAGGTTAAGTCGCCCTCGCTCGAGCGAGGGCGACTTAACCTTAGGA |
| shTUFM (#2) | F | CCGGGAGGACCTGAAGTTCAACCTACTCGAGTAGGTTGAACTTCAGGTCCTCTTTTT |
|  | R | AATTAAAAAGAGGACCTGAAGTTCAACCTACTCGAGTAGGTTGAACTTCAGGTCCTC |
| CASP9  gRNA | F | CACCGACCAGAGATTCGCAAACCAG  AAACCTGGTTTGCGAATCTCTGGTC  AAACCTGGTTTGCGAATCTCTGGTC |
|  | R | AAACCTGGTTTGCGAATCTCTGGTC |
| ATG7  gRNA | F | CACCGGAAGCTGAACGAGTATCGGC |
|  | R | AAACGCCGATACTCGTTCAGCTTCC  CACCGGTCTTCCGCTGCAGTTTCCC |
| ATG12  gRNA | F  R | CACCGGTCTTCCGCTGCAGTTTCCC |
|  | R | AAACGGGAAACTGCAGCGGAAGACC |

**Table S3 Antibodies used in the study**

| **ANTIBODIES** | **SOURCE** | **IDENTIFIER** |
| --- | --- | --- |
| Rabbit polyclonal anti-LDH (H-160) | Santa Cruz Biotechnology | sc-33781 |
| Mouse monoclonal anti-GST (B-14) | Santa Cruz Biotechnology | sc-138 |
| Mouse monoclonal anti-HSP60 (B-9) | Santa Cruz Biotechnology | sc-271215 |
| Mouse monoclonal anti-TOM20 (F-10) | Santa Cruz Biotechnology | sc-17764 |
| Mouse monoclonal anti-TFAM | Santa Cruz Biotechnology | sc-376672 |
| Mouse monoclonal anti-FLOT1 (C-2) | Santa Cruz Biotechnology | sc-74566 |
| Mouse monoclonal anti-EF-Tu/TUFM (A-5) | Santa Cruz Biotechnology | sc-393924 |
| Rabbit polyclonal anti-MCL-1 (S-19) | Santa Cruz Biotechnology | sc-819 |
| Mouse monoclonal anti-MAVS (E-3) | Santa Cruz Biotechnology | sc-166583 |
| Mouse monoclonal anti-NRLX1 (also known as NOD9) (F-2) | Santa Cruz Biotechnology | sc-374514 |
| Mouse monoclonal anti-V5 tag | Invitrogen | R960-25 |
| Mouse monoclonal anti-Flag tag (M2) | Sigma | F3165 |
| Rat monoclonal anti-HA tag (3F10) | Sigma | 11867423001 |
| Mouse monoclonal Anti-β-Actin | Proteintech | 60008-1-lg |
| HRP-conjugated T7 Tag | Novagen (Fisher Scientific) | 69-048-3MI |
| Rabbit monoclonal anti-ATG12 (D88H11) | Cell Signaling Technology | #4180 |
| Rabbit monoclonal anti-Cytochrome c (D18C7) | Cell Signaling Technology | #11940 |
| Mouse monoclonal Caspase-8 (1C12) | Cell Signaling Technology | #9746 |
| Rabbit monoclonal Cleaved Caspase-3 (D175) (5A1E) | Cell Signaling Technology | #9664 |
| Rabbit monoclonal PARP (46D11) | Cell Signaling Technology | #9532 |
| Rabbit monoclonal anti-VDAC (D73D12) | Cell Signaling Technology | #4661 |
| Rat polyclonal anti-DYKDDDDK tag (L5) gel | BioLegend | 651502 |
| Rabbit monoclonal anti-ADSL | Sino Biological | 11287-R007 |
| Goat polyclonal anti-V5 agarose | Bethyl Laboratories | S190-119 |
| Purified goat IgG-agarose | Bethyl Laboratories | S50-100 |
| Rabbit polyclonal anti-vIRF-1 | A gift from Dr. Gary Hayward | N/A |
| Goat anti-Mouse 488 | Invitrogen | A-11029 |
| Goat anti-Rabbit 594 | Invitrogen | A-11037 |
